# Prognostic impact of Skp2, ER and PGR in male and female patients with soft tissue sarcomas

**DOI:** 10.1186/1472-6890-13-9

**Published:** 2013-03-15

**Authors:** Sveinung W Sorbye, Thomas K Kilvaer, Andrej Valkov, Tom Donnem, Eivind Smeland, Khalid Al-Shibli, Roy M Bremnes, Lill-Tove Busund

**Affiliations:** 1 Department of Clinical Pathology, University Hospital of North Norway, Tromso, Norway; 2 Institute of Medical Biology, University of Tromso, Tromso, Norway; 3 Department of Oncology, University Hospital of North Norway, Tromso, Norway; 4 Institute of Clinical Medicine, University of Tromso, Tromso, Norway; 5 Department of Pathology, Nordland Central Hospital, Bodo, Norway

## Abstract

**Background:**

S-phase kinase-associated protein 2 (Skp2) is a member of mammalian F-box proteins. The purpose of this study is to clarify the prognostic significance of expression of Skp2 related to gender, estrogen receptor (ER) and progesterone receptor (PGR) in soft tissue sarcomas (STS). Skp2 has been demonstrated to display an oncogenic function since its overexpression has been observed in many human cancers. Optimized treatment of STS requires better identification of high-risk patients who will benefit from adjuvant therapy. The prognostic significance of Skp2 related to ER and PGR in STS has not been sufficiently investigated.

**Methods:**

Tissue microarrays from 193 STS patients were constructed from duplicate cores of viable and representative neoplastic tumor areas. Immunohistochemistry was used to evaluate the expression of Skp2, ER and PGR.

**Results:**

In univariate analyses, high tumor expression of Skp2 correlated (p = 0.050) with reduced disease-specific survival (DSS). In subgroup analyses expression of PGR in males (p = 0.010) and in patients older than 60 years (p = 0.043) were negative prognostic factors for DSS. Expression of ER in females was a positive prognostic factor for DSS (p = 0.041). In co-expression analyses in the whole cohort, low expression of Skp2 in combination with low expression of ER was positive for DSS (p = 0.049). In females high expression of Skp2 in combination with low expression of ER was a negative prognosticator (p = 0.021). In the multivariate analyses, age (p = 0.012), malignancy grade (p < 0.001), wide resection margins (P = 0.010), ER negative / PGR positive co-expression profile (p = 0.002) and ER positive / PGR negative co-expression profile (p = 0.015) were independent negative prognostic factors for DSS. In females expression of Skp2 (p = 0.006) was associated with shorter DSS.

**Conclusions:**

We found diverse prognostic impacts of expression of Skp2, ER, PGR and DSS in male and female patients with STS. In men, but not women, ER positive / PGR negative co-expression profile was an independent negative prognostic factor for DSS. In women, but not men, high expression of Skp2 was associated with reduced DSS.

## Background

S-phase kinase-associated protein 2 (Skp2), a mammalian F-box protein, displays S-phase-promoting function, through ubiquitin-mediated proteolysis of the CDK inhibitor p27. Skp2 has been shown to regulate cellular proliferation by targeting several cell cycle-regulated proteins for ubiquitination and degradation. Skp2 has also been demonstrated to display an oncogenic function since its overexpression has been observed in many human cancers [1]. High expression of Skp2 was reported to correlate with reduced overall survival in patients with myxofibrosarcoma [2,3]. Di Vizio et al. [4] found that Skp2 expression correlates with poor prognosis in gastrointestinal stromal tumors (GIST). Oliveira found that Skp2 expression is associated with cell proliferation and a worse prognosis in 182 soft tissue sarcomas [5]. In a previous study we showed that high expression of Skp2 was a negative prognostic factor for DSS [6]. Interestingly, this correlation was statistically significant in females only, not in males. This may be related to differences in expression of sexual hormone receptors (ER and PGR) in male and female STS patients [7,8]. In previous studies, we have shown the prognostic value of female steroid hormone receptors in STSs, both alone and in coexpression with TGF-β, fascin and Akt isoforms [7-9]. Such prognostic impact is not surprising, since both ER and PGR regulate growth and cell differentiation upon ligand-dependent and ligand-independent activation and are in essence growth factors. However, the prognostic significance of Skp2 related to ER and PGR in STS has not been sufficiently investigated.

The purpose of this study is to clarify the prognostic significance of expression of Skp2 related to age, gender and female steroid hormone receptors (ER and PGR) in non-gastrointestinal stromal tumor (non-GIST) STS. To achieve this, we analyzed the expression of these markers in 193 patients with non-GIST STS in relation to demographic and other clinicopathological variables. Our major hypothesis is that a different prognostic significance of Skp2 in men and women exists and is related to diverse gender expressions of ER and PGR.

## Methods

Primary tumor tissues from patients diagnosed with STS at the University Hospital of North Norway (UNN) from 1973 to 2006 and the Hospitals of Arkhangelsk region, Russia, were used in this retrospective study. In total, 496 potentially suitable patient records were identified from the hospitals’ databases. Of these, 247 patients were excluded due to missing clinical data (n = 86) or inadequate material for histological examination (n = 161). In addition, 33 were excluded because of metastasis at the time of the diagnosis, 13 were excluded because they had no surgery, and 10 patients had both metastasis and no surgery, leaving a total of 193 patients eligible for this study. This report includes data for 131 Norwegian patients and 62 Russian patients followed until September 2009. The median follow-up was 38 (range 0–392) months. Complete demographic and clinical data were collected retrospectively. Formalin-fixed and paraffin-embedded tumor specimens were obtained from the archives of the Departments of Pathology at UNN and Arkhangelsk. The tumors were graded according to the French Fédération Nationales des Centres de Lutte Contre le Cancer (FNCLCC) system [WHO Tumors of Soft Tissue and bone, 2002]. Wide resection margins were defined as wide local resection with free microscopic margins or amputation of the affected limb or organ. Non-wide resection margins were defined as either marginal or intralesional resection margins.

### Microarray construction

Two pathologists (AV and SWS) reviewed the histology of all soft tissue sarcoma cases. Tissue microarrays (TMAs) were constructed for high-throughput molecular pathology research [10]. The most representative areas of viable tumor cells were carefully selected and marked on the hematoxylin and eosin (HE) slides for the corresponding donor blocks and sampled for the tissue microarray collector blocks. The TMAs were assembled using a tissue-arraying instrument (Beecher Instruments).

Studies suggest that punching multiple 0.6 mm cores from different regions captures the heterogeneity of the tumors more accurately than a single 2 to 4 mm core [11]. We therefore chose to use two 0.6-mm cores of viable neoplastic tissue. After reviewing all original sections of the tumor and taking heterogeneity into consideration, the two cores were selected to be as representative as possible (different areas). To include all core samples, 12 tissue array blocks were constructed. Multiple 4-μm sections were cut with a Micron microtome (HM355S) and stained with specific antibodies for immunohistochemistry (IHC).

### Immunohistochemistry (IHC)

The applied antibodies were subjected to in-house validation by the manufacturer of IHC analysis on paraffin-embedded material. All staining was performed in the Ventana Benchmark XT automated slide stainer (Ventana Medical System, Illkirch, France). Before staining, the sections were incubated over night at 60 degrees Celsius. Tissue sections were incubated with primary mouse monoclonal antibodies recognizing Skp2 (Zymed, catalog number 18–0307, 1:10), ER (Ventana, catalog number 790–4324, ready to use) and PGR (Ventana, catalog number 790–4296). The incubation periods were 40 minutes for Skp2, 32 minutes for ER and 24 min for PGR. This was followed by application of liquid diaminobenzidine as substrate-chromogen, yielding a brown reaction product at the site of the target antigen (Ventana iView DAB Detection Kit, catalog number 760–091). iVIEW DAB Detection Kit is an indirect biotin streptavidin system for detecting mouse and rabbit primary antibodies. The DAB chromogen produces a dark brown precipitate that is readily visualized by light microscopy. All reagents are provided pre-diluted by the manufacturer for use in Ventana Benchmark XT. Finally, slides were counterstained with hematoxylin to visualize the nuclei. For each antibody, including negative controls, all TMA staining were performed in a single experiment. In the TMA we also used cores from carcinomas and normal tissue as positive and negative controls.

### Scoring of IHC

**Figure 1:**
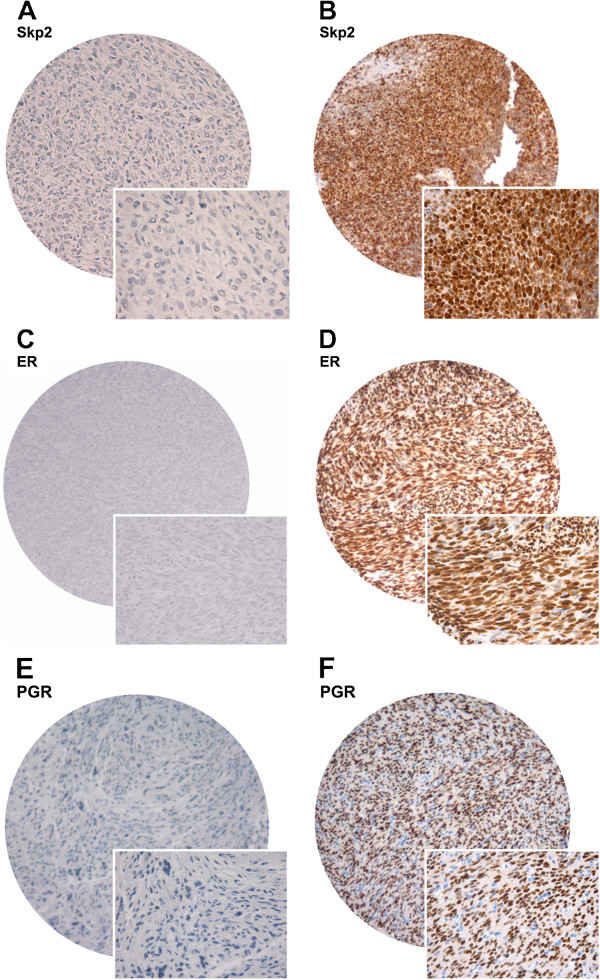
**Pictures of cores.** Immunohistochemistry microscopic pictures of tissue micro array of soft tissue sarcoma representing different expression of Skp2 and ER. (**A**) Skp2 low score; (**B**) Skp2 high score; (**C**) ER low score; (**D**) ER high score; (**E**) PGR low score; (**F**) PGR high score; Original magnification ×100 and ×400.

The ARIOL imaging system (Genetix, San Jose, CA) was used to scan the slides for antibody staining of the TMAs. The specimens were scanned at a low resolution (1.25×) and a high resolution (20×) using an Olympus BX 61 microscope with an automated platform (Prior). The slides were loaded in the automated slide loader (Applied Imaging SL 50). Representative and viable tissue sections were scored manually on a computer screen semi-quantitatively for nuclear and/or cytoplasmic staining. The expression of Skp2, ER and PGR was scored as: 0, negative; 1, weak; 2, intermediate and 3, strong (Figure 1). The score for each patient was based on the mean scoring of cores from one or several biopsies. To achieve maximal reproducibility in all cases, every staining was dichotomized (negative and positive expression). Positive expression was defined as mean score > 0. All samples were anonymized and independently scored by two pathologists (AV and SWS). In case of disagreement, the slides were re-examined and the observers reached a consensus. When assessing a variable for a given score, the scores of the other variables and the outcome were hidden from the observers.

### Statistical methods

All statistical analysis was performed using the statistical package SPSS (Chicago, IL), version 18. The IHC scores from each observer were compared for inter-observer reliability by use of a two-way random effects model with absolute agreement definition. The intra-class correlation coefficient (reliability coefficient) was obtained from these results.

Chi-square and Fisher exact tests were used to examine the association between molecular marker expression and various clinicopathological parameters. Univariate analyses were done using the Kaplan-Meier method, and statistical significance between survival curves was assessed by the log rank test. Disease-specific survival (DSS) was determined from the date of histologically confirmed STS diagnosis. Correlation of marker expression was done using the Pearson correlation (2-tailed) at the 0.05 and 0.01 levels.

Multivariate analysis was carried out using the Cox proportional hazards model to assess the specific impact of each pre-treatment variable on survival in the presence of other variables. Variables of significant value from the univariate analysis were entered into the Cox regression analysis. Probability for stepwise entry and removal was set at 0.05 and 0.10, respectively. The significance level used was p < 0.05.

### Consent

The National Cancer Data Inspection Board and The Regional Committee for Research Ethics (REK nord) approved the study. The material was collected from our approved biobank for paraffin embedded material and slides. The Regional Committee approved that written consent from the patients for their information to be stored in the hospital database and used for research was not needed because most of the material was more than 10 years old, and most of the patients being dead. The ethics committee specifically waived the need for consent. Data were analyzed anonymously.

## Results

### Clinicopathological variables

Demographic, clinical, and histopathological variables are shown in Table 1. Patient age ranged from 0–89 years (mean 55 years), and 42% of patients (81/193) were male. Treatment for all patients included surgery: 104 patients received surgery only; 52 patients received surgery and radiotherapy; 28 patients received surgery and chemotherapy; 9 patients received surgery, radiotherapy and chemotherapy. The 5-year survival for patients with wide and non-wide resection margins was 66% and 46% respectively, Table 1.

**Table 1:** Prognostic clinicopathological variables as predictors for disease-specific survival of soft tissue sarcomas (univariate analysis, log rank test), N = 193

**Characteristic**	**Patients (n)**	**Patients (%)**	**Median survival (months)**	**5-Year survival (%)**	**P**
**Age**					
<20 years	17	9	190	47	0.064
20–59 years	85	44	235	63	
≥60 years	91	47	111	51	
**Gender**					
Male	81	42	235	60	0.087
Female	112	58	180	53	
**Nationality**					
Norwegian	131	68	228	62	0.005
Russian	62	32	81	44	
**Histology**					
Pleomorphic sarcoma	57	30	52	45	0.031
Leiomyosarcoma	47	24	89	64	
Liposarcoma	32	17	NR	71	
MF/MFT	16	8	123	56	
Angiosarcoma	8	4	10	38	
Rhabdomyosarcoma	9	5	NR	67	
MPNST	9	5	NR	56	
Synovial sarcoma	12	6	31	30	
Other STS	3	2	NR	-	
**Tumor localization**					
Extremities	78	40	201	56	0.922
Trunk	37	19	214	53	
Retroperitoneum	27	14	135	51	
Head/Neck	13	7	191	58	
Visceral	38	20	202	62	
**Tumor size**					
<5 cm	57	30	257	69	0.026
5–9 cm	73	38	183	54	
≥10 cm	61	32	127	48	
Missing	2	1			
**Malignancy grade FNCLCC**					
1	54	28	NR	81	<0.001
2	76	39	80	55	
3	63	33	28	36	
**Surgical margins**					
Wide	97	50	254	66	<0.001
Non-wide	96	50	128	46	
**Chemotherapy**					
No	156	81	207	57	0.669
Yes	37	19	180	51	
**Radiotherapy**					
No	132	68	216	58	0.190
Yes	61	32	152	52	

*Abbreviations: MF/MFT*, malignant fibroblastic/myofibroblastic tumors; *MPNST*, malignant peripheral nerve sheath tumor; *STS*, soft tissue sarcomas; *NR*, not reached; *NOS*, non specified.

### Inter-observer variability

There was good scoring agreement between the two investigating pathologists. The IHC scores from each observer were compared using a two-way random effects model with absolute agreement definition. The intra-class correlation coefficients (reliability coefficients, r) obtained from these results were 0.94 for Skp2 (p < 0.001), 0.92 for ER (p < 0.001) and 0.96 for PGR (p < 0.001).

### Univariate analyses

Nationality, histology, tumor size, malignancy grade and surgical margins were all significant indicators for disease-specific survival (DSS) in univariate analyses (Table 1). Table 2 shows the percentage of high expression of ER, PGR and Skp2 in the different histological subtypes. Chi-square test showed no differences in overall expression of ER, PGR and Skp2 with respect to the different histological subtypes.

**Table 2:** Percentage of high expression of ER, PGR and Skp2 in the different histological subtypes N = 193

**Histology**	**N**	ER (%)*	PGR (%)**	Skp2 (%)***
Pleomorphic sarcoma	57	40	26	37
Leiomyosarcoma	47	50	43	40
Liposarcoma	32	35	23	21
MF/MFT	16	27	29	36
Angiosarcoma	8	25	13	29
Rhabdomyosarcoma	9	50	56	67
MPNST	9	11	11	44
Synovial sarcoma	12	40	27	50
Other STS	3	67	33	67
Total	193	39	30	38

* Chi 8.516, p = 0.385.

** Chi 10.238, p = 0.249.

*** Chi 8.596, p = 0.377.

*Abbreviations: MF/MFT*, malignant fibroblastic/myofibroblastic tumors; *MPNST*, malignant peripheral nerve sheath tumor.

Chi-square test showed no differences in percentage of high expression of ER, PGR and Skp2 in the different histological subtypes.

**Figure 2:**
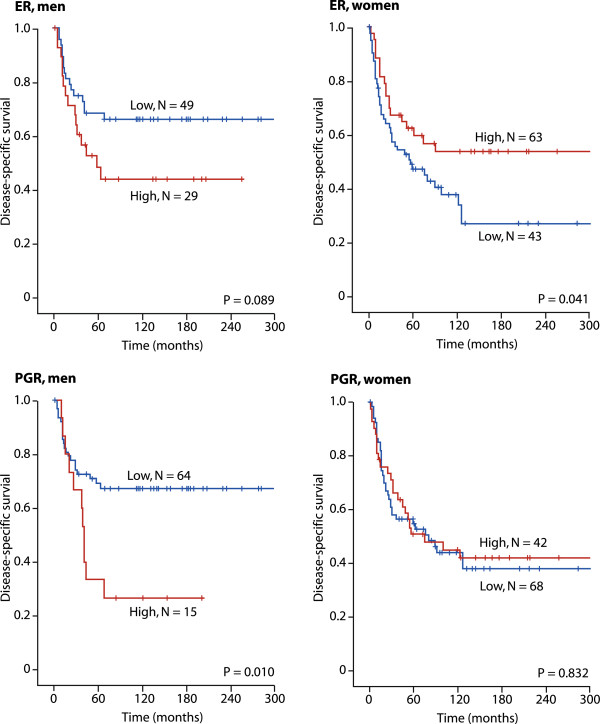
**Survival plots ER and PGR.** Disease-specific survival curves for high and low expression of ER and PGR in male (N = 81) and female (N = 112) patients with soft tissue sarcomas.

In univariate analyses, increased expression of Skp2 (p = 0.050) correlated significantly with reduced DSS, (Table 3 and Figure 2). No such relationship was apparent for ER and PGR when males and females were combined in one group.

**Table 3:** Expression of markers, gender and their prediction for disease-specific survival in patients with soft tissue sarcomas (univariate analysis; log-rank test), All = 193, Males = 81, Females = 112

**Marker expression**	**Patients (n)**	**Patients (%)**	**Median survival (months)**	**5-year survival (%)**	**P**
**Skp2, all**					
Low	109	56	NR	63	0.050
High	67	45	59	50	
Missing	17	9			
**Skp2, men**					
Low	50	62	NR	63	0.577
High	23	28	67	61	
Missing	8	10			
**Skp2, women**					
Low	59	53	NR	63	0.066
High	44	39	49	44	
Missing	9	8			
**ER, all**					
Low	112	58	123	57	0.725
High	72	67	91	57	
Missing	9	5			
**ER, men**					
Low	49	60	NR	69	0.089
High	29	36	58	49	
Missing	3	4			
**ER, women**					
Low	63	56	57	47	0.041
High	43	38	NR	62	
Missing	6	5			
**PGR, all**					
Low	132	68	NR	62	0.101
High	57	30	52	46	
Missing	4	2			
**PGR, men**					
Low	64	79	NR	69	0.010
High	15	19	41	33	
Missing	2	2			
**PGR, women**					
Low	68	61	80	55	0.832
High	42	38	74	51	
Missing	2	2			

*Abbreviations: NR*, not reached.

In subgroup analyses (Tables 3 and 4), increased PGR expression in men (p = 0.010) and in patients older than 60 years (p = 0.043) was associated with a reduced DSS. Increased ER expression in women was associated with longer DSS (p = 0.041). High expression of ER were associated with favorable survival in patients with rhabdomyosarcoma (N = 9, p = 0.040). High expression of ER was associated with poor survival in patients with synovial sarcoma (N = 12, p = 0.010). There were no significant differences in survival according to high or low expression of Skp2 in any of the histological subtypes (data not shown).

**Table 4:** Expression of markers, age and their prediction for disease-specific survival in patients with soft tissue sarcomas (univariate analysis; log-rank test)

**Marker expression**	**Patients (n)**	**Patients (%)**	**Median survival (months)**	**5-Year survival (%)**	**P**
**Skp2, <60 years, N = 99**					
Low	52	53	NR	71	0.074
High	38	38	67	56	
Missing	9	9			
**Skp2, ≥60 years, N = 94**					
Low	57	61	80	57	0.188
High	29	31	36	42	
Missing	8	9			
**ER, <60 years, N = 99**					
Low	55	56	127	59	0.197
High	40	40	NR	67	
Missing	4	4			
**ER, ≥60 years, N = 94**					
Low	57	61	80	55	0.293
High	32	34	52	44	
Missing	5	5			
**PGR, <60 years, N = 99**					
Low	63	64	NR	67	0.488
High	34	34	NR	55	
Missing	2	2			
**PGR, ≥60 years, N = 94**					
Low	69	73	91	57	0.043
High	23	24	39	32	
Missing	2	2			

*Abbreviations: NR*, not reached.

In patients with low expression of ER (N = 112), men had better 5-year survival (69%) compared to women (47%, p = 0.002), while there were no differences (p = 0.376) between men and women in patients with high expression of ER (N = 72). In patients with low expression of PGR (N = 132), men had better 5-year survival (69%) compared to women (55%, p = 0.013), while there were no differences (p = 0.271) between men and women in patients with high expression of PGR (N = 57). There were no differences in survival between men and women in univariate analyses of patients with low (N = 109, p = 0.529) or high (N = 67, p = 0.233) expression of Skp2 (data not shown).

**Figure 3:**
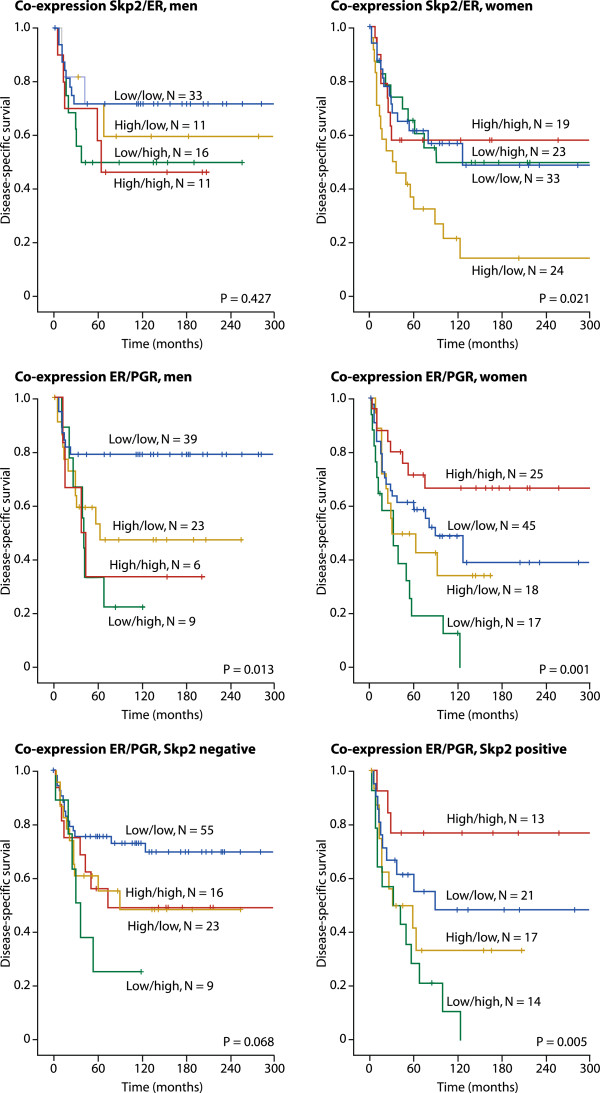
**Survival plots co-expression.** Disease-specific survival curves for co-expression of Skp2, ER or PGR in males (N = 81), females (N = 112) and co-expression of ER and PGR in Skp2 negative (N = 109) and Skp2 positive (N = 67) patients.

In co-expression analyses (Table 5) Skp2 negative / ER negative profile was associated with longer DSS (p = 0.049). In women a Skp2 positive and ER negative profile was associated with reduced DSS (p = 0.021), Table 5 and Figure 3. In men a double negative ER/PGR profile was associated with longer DSS (p = 0.013) while in women a double positive ER/PGR was associated with longer DSS (p = 0.001). In patients younger than 60 years the combination ER negative and PGR positive was associated with shorter DSS. In the whole cohort of patients a triple positive expression of ER, PGR and Skp2 was associated with longer DSS (p = 0.005), Figure 3. Triple negative expression of ER, PGR and Skp2 was also associated with longer DSS, but not statistically significant (p = 0.068), Figure 3. ER negative / PGR positive co-expression was associated with shorter DSS regardless of Skp2 expression, Table 6.

**Table 5:** Co-expression of Skp2/ER, Skp2/PGR and their prediction for disease-specific survival in patients with soft tissue sarcomas (univariate analysis; log-rank test), All = 193, Men = 81, Women = 112

**Co-expression**	**Patients (n)**	**Patients (%)**	**Median survival (months)**	**5-Year survival (%)**	**P**
**Skp2 / ER, all**					
Low/low	66	34	NR	67	0.049
Low/high	39	20	91	59	
High/low	35	18	57	44	
High/high	30	16	NR	58	
Missing	23	12			
**Skp2 / ER, men**					
Low/low	33	41	NR	72	0.427
Low/high	16	20	37	50	
High/low	11	14	NR	72	
High/high	11	14	63	58	
Missing	10	12			
**Skp2 / ER, women**					
Low/low	33	29	127	61	0.021
Low/high	23	21	91	65	
High/low	24	21	31	32	
High/high	19	17	NR	58	
Missing	13	12			
**Skp2 / PGR, all**					
Low/low	80	41	NR	71	0.056
Low/high	25	13	54	46	
High/low	40	21	59	49	
High/high	27	14	67	51	
Missing	21	11			
**Skp2 / PGR, men**					
Low/low	41	51	NR	73	0.141
Low/high	7	9	26	29	
High/low	18	22	NR	61	
High/high	5	6	67	60	
Missing	10	12			
**Skp2 / PGR, women**					
Low/low	39	35	NR	68	0.234
Low/high	18	16	75	54	
High/low	22	20	29	39	
High/high	22	20	57	49	
Missing	11	10			

*Abbreviations: NR*, not reached.

**Table 6:** Co-expression of ER/PGR and their prediction for disease-specific survival in patients with soft tissue sarcomas (univariate analysis; log-rank test)

**Co-expression**	**Patients (n)**	**Patients (%)**	**Median survival (months)**	**5-year survival (%)**	**P**
**ER / PGR, all, N = 193**					
Low/low	84	44	NR	69	<0.001
Low/high	26	13	38	24	
High/low	41	21	62	52	
High/high	31	16	NR	64	
Missing	11	6			
**ER / PGR, men, N = 81**					
Low/low	39	48	NR	79	0.013
Low/high	9	11	41	33	
High/low	23	28	63	53	
High/high	6	7	37	33	
Missing	4	5			
**ER / PGR, women, N = 121**					
Low/low	45	40	89	59	0.001
Low/high	17	15	31	19	
High/low	18	16	29	50	
High/high	25	22	NR	72	
Missing	7	6			
**ER / PGR, <60 years, N = 99**					
Low/low	41	41	NR	72	0.001
Low/high	13	13	31	23	
High/low	19	19	NR	58	
High/high	21	21	NR	76	
Missing	5	5			
**ER / PGR, ≥60 years, N = 94**					
Low/low	43	46	NR	64	0.052
Low/high	13	14	39	26	
High/low	22	23	58	47	
High/high	10	11	37	40	
Missing	6	6			
**ER / PGR, Skp2 low, N = 109**					
Low/low	55	50	NR	76	0.068
Low/high	9	8	68	25	
High/low	23	21	91	61	
High/high	16	15	75	56	
Missing	6	6			
**ER / PGR, Skp2 high, N = 67**					
Low/low	21	31	89	55	0.005
Low/high	14	21	31	29	
High/low	17	25	29	42	
High/high	13	19	NR	77	
Missing	2	3			

*Abbreviations: NR*, not reached.

Taking into consideration the possible distortion of results by gender-related sarcomas (i.e. leiomyosarcoma in uterus) we have attempted to exclude these sarcomas and recalculate all analyses. There were no significant differences in the results compared to those obtained without exclusion of gender-related sarcomas (data not shown).

### Multivariate analyses

Significant demographic, clinicopathological and expression variables from the univariate analyses were entered into the multivariate Cox regression analysis (Table 7). In the multivariate analyses, age (p = 0.012), malignancy grade (p < 0.001), wide resection margins (p = 0.010), ER negative / PGR positive co-expression (p = 0.002) and ER positive / PGR negative co-expression (p = 0.015) were independent negative prognostic factors for DSS. In women, expression of Skp2 (p = 0.006) was associated with reduced DSS. In women, tumor size (p = 0.020) and nationality (p = 0.014) were independent prognostic factors for DSS, Table 7. In multivariate analyses co-expression of Skp2/ER or Skp2/PGR were not stronger prognosticators for DSS than single expression of Skp2, ER and PGR (data not shown).

**Table 7:** Results of Cox regression analysis summarizing prognostic factors in patients with soft tissue sarcomas

	**All patients, N = 193**			**Men, N = 81**			**Women, N = 112**		
**Factor**	**Hazard ratio**	**95% CI**	**P**	**Hazard ratio**	**95% CI**	**P**	**Hazard ratio**	**95% CI**	**P**
**Age**									
0–59 years	1.00			1.00			1.00		
≥60 years	1.84	1.15-2.95	0.012	1.69	0.65-4.41	0.282	1.51	0.83-2.77	0.179
**Nationality**									
Norwegian	1.00			1.00			1.00		
Russian	1.49	0.88-2.52	0.143	1.39	0.41-4.66	0.598	2.51	1.20-5.21	0.014
**Tumor size**									
<5 cm	1.00		0.138*	1.00		0.668*	1.00		0.020*
5–9 cm	1.47	0.79-2.73	0.226	1.68	0.54-5.25	0.372	1.71	0.77-3.77	0.187
≥10 cm	1.91	1.01-3.60	0.047	1.32	0.40-4.39	0.652	3.14	1.38-7.15	0.006
**Malignancy grade FNCLCC**									
1	1.00		<0.001*	1.00		<0.001	1.00		0.004*
2	2.72	1.36-5.46	0.005	3.07	0.86-10.96	0.084	4.33	1.76-10.67	0.001
3	4.61	2.26-9.40	<0.001	15.47	4.36-54.97	<0.001	4.23	1.64-10.89	0.003
**Resection margins**									
Wide	1.00			1.00			1.00		
Non-wide	1.87	1.16-3.02	0.010	7.69	2.67-22.16	<0.001	0.81	0.42-1.54	0.512
**Skp2**									
Low	1.00			1.00			1.00		
High	1.48	0.87-2.52	0.151	0.46	0.19-1.12	0.088	2.52	1.31-4.85	0.006
**ER / PGR**									
Low/low	1.00		0.006*	1.00		0.004*	1.00		0.216*
Low/high	2.64	1.43-4.85	0.002	4.99	1.31-18.97	0.018	1.91	0.89-4.11	0.097
High/low	2.07	1.15-3.73	0.015	8.35	2.55-27.36	<0.001	1.76	0.79-3.93	0.170
High/high	1.16	0.57-2.38	0.682	4.50	0.96-21.13	0.056	0.92	0.36-2.35	0.868

* Overall significance as a prognostic factor. The difference between the individual p-value and total p-value in the multivariate analysis is relevant in cases where there are more than two categories for a given variable. Overall p-value is calculated based on a general assessment of all categories for the given variable, but the individual p-value only calculates the significance of a given category versus the reference category.

## Discussion

In this large-scale study, we evaluated the prognostic significance of expression of Skp2 related to age, gender, ER and PGR in 193 STS patients. Our hypothesis was confirmed. We found diverse prognostic DSS impacts from gender related expression of Skp2, ER, PGR and DSS in STS. In men, but not women, an ER positive/PGR negative co-expression profile was an independent negative prognostic factor for DSS. In women, but not men, high expression of Skp2 was associated with reduced DSS. High expression of ER reduced the negative impact of Skp2 in women. While women with the Skp2 positive / ER positive phenotype had favorable survival, women with the Skp2 positive / ER negative phenotype had poor survival. To the best of our knowledge, this is the first prognostic evaluation of Skp2 related to the female hormone receptors ER and PGR in STS.

Expression of ER and PGR is a routinely investigated indicator of endocrine therapy success in breast cancer [12,13] and a modest, but significantly better overall survival of anti-estrogen receptor therapy has been documented [14]. ER and PGR are also reported to be positive prognosticators of uterine leiomyosarcomas [15]. However, extra-uterine sarcomas have barely been explored in this context. The distribution and prognostic value of expression of these steroid hormone receptors in STS are therefore of great scientific interest. In our study, in the univariate analyses, ER showed a significantly favorable influence on survival in female patients, but not in males. PGR was an unfavorable prognosticator for men, but not for women. In multivariate analysis ER positive / PGR negative co-expression is an independent negative prognostic factor for DSS in males, but not in females.

We have modified the Allred score for STS and used 1% positivity as cut-off value [7,16]. The strong and moderate (score 3 and 2, respectively) hormone receptor expression occurred mostly in sarcomas of uterus, pelvis and breast, while the weak (score 1) expression of both ER and PGR was surprisingly evenly distributed among location, gender and age. Generally, 39% of the tumors expressed ER and 30% expressed PGR in our material. Roughly half of the patients expressed at least one of these receptors. The findings are in partial agreement with findings of Chaudhuri et al. [17] who found ER to be positive in 24% of STS.

Huang et al. suggested that the therapeutic strategies designed to reduce Skp2 may play an important clinical role in treatment of breast cancer cells, especially ER/HER2 negative breast cancers [18]. Voduc et al. found cyclin E and Skp2 to be prognostic for breast cancer-specific survival in univariate analyses. Double positive expression of cyclin E / Skp2 was associated with young age at diagnosis, grade 3 tumors, ER-negative status and HER2 negative status [19]. Zheng et al. found that higher levels of Skp2 were detected more frequently in ER-negative breast cancer tumors and tumors metastatic to the axillary lymph nodes [20]. Signoretti et al. also found that higher levels of Skp2 are present more frequently in ER-negative tumors than in ER-positive cases. The subset of Skp2 positive / ER negative breast carcinomas were also characterized by high tumor grade and HER2 negative [21]. In our material, the five year DSS in Skp2 positive / ER negative women with STS was 32% compared to 58% in Skp2 positive / ER positive women (P = 0.021).

In our previous work we have shown that ER and PGR expression possess variable prognostic significance depending on gender, both *per se* and in co-expression with TGF-β, fascin and Akt isoforms [7-9]. In the present study, the prognostic diversity of Skp2, ER and PGR in men and women was seen in the different co-expression profiles: female patients with Skp2 positive / ER negative profile had decreased survival rates. For men, the Skp2 negative / ER negative profile was the most favorable phenotype. PGR expression in men, but not women, was associated with a shorter DSS. ER expression in women, but not men, was associated with a longer DSS. The ER negative / PGR positive profile was a significantly unfavorable factor for the whole patient cohort both in univariate and multivariate analysis. Interestingly, such a profile occurred in only 2% of patients in one large-scale study based on 3000 breast cancer cases [22], while in our STS study this profile was seen in 13% of tumors.

The data collection introduced problems in identifying adequate numbers of similar patients with similar tumors and with the same treatment traditions. These are well known problems when conducting STS studies. Our findings are in large hypothesis generating, and to be more conclusive future STS studies must be based on large, multi-institutional and multinational studies with possibilities to establish adequately sized STS patient cohorts of homogenous tumor groups. However, all tumors investigated herein had mesenchymal derivation and belong to the same generic group.

## Conclusions

In conclusion, there were different prognostic impacts of expression of Skp2, ER, PGR and DSS in male and female patients with STS. In men, but not in women, ER positive / PGR negative co-expression was an independent negative prognostic factor for DSS. In women, but not in men, expression of Skp2 was associated with reduced DSS.

## Competing interests

The authors declare that they have no competing interests.

## Authors’ contributions

SWS, TK, AV, TD, RMB and LTB participated in the design of the study. TK, ES and AV collected clinical information. SWS and AV reviewed all the histological diagnosis, histological grading, selected and marked the slides for TMA construction. SWS, TK and AV performed the experiments. SWS, TK, AV, TD, RMB and LTB performed the statistical analysis. SWS, TK, AV, TD, ES, KAS and LTB contributed reagents/materials/analysis tools. SWS, TD, ES, KAS, RMB and LTB drafted the manuscript. All authors read and approved the final manuscript.

## Pre-publication history

The pre-publication history for this paper can be accessed here:

http://www.biomedcentral.com/1472-6890/13/9/prepub
